# Jugular venous reflux and brain parenchyma volumes in elderly patients with mild cognitive impairment and Alzheimer’s disease

**DOI:** 10.1186/1471-2377-13-157

**Published:** 2013-10-31

**Authors:** Clive Beggs, Chih-Ping Chung, Niels Bergsland, Pei-Ning Wang, Simon Shepherd, Chun-Yu Cheng, Michael G Dwyer, Han-Hwa Hu, Robert Zivadinov

**Affiliations:** 1 Medical Biophysics Laboratory, School of Engineering, Design & Technology, University of Bradford, Bradford, West Yorkshire BD7 1DP, UK; 2 Department of Neurology, Taipei Veterans General Hospital, Taipei, Taiwan; 3 Department of Neurology, National Yang Ming University of Medicine, Taipei, Taiwan; 4 Buffalo Neuroimaging Analysis Center, Department of Neurology, University at Buffalo, Buffalo, NY, USA; 5 Institute of Brain Science, National Yang-Ming University School of Medicine, Taipei, Taiwan

**Keywords:** Jugular venous reflux, Healthy controls, Mild cognitive impairment, Alzheimer's disease, Brain parenchyma volume

## Abstract

**Background:**

To determine whether or not jugular venous reflux (JVR) is associated with structural brain parenchyma changes in individuals with mild cognitive impairment (MCI) and Alzheimer’s disease (AD).

**Methods:**

16 AD patients (mean (SD): 81.9 (5.8) years), 33 MCI patients (mean (SD): 81.4 (6.1) years) and 18 healthy elderly controls (mean (SD): 81.5 (3.4) years) underwent duplex ultrasonography and magnetic resonance imaging scans to quantify structural brain parenchyma changes. Normalized whole brain (WB), gray matter (GM) and white matter (WM) volumes were collected, together with CSF volume.

**Results:**

JVR was strongly associated with increased normalized WB (*p* = 0.014) and GM (*p* = 0.002) volumes across all three subject groups. There was a trend towards increased WB and GM volumes, which was accompanied by decreased CSF volume, in the JVR-positive subjects in both the MCI and AD groups. When the MCI and AD subjects were aggregated together significant increases were observed in both normalized WB (*p* = 0.009) and GM (*p* = 0.003) volumes for the JVR-positive group. No corresponding increases were observed for the JVR-positive subjects in the control group. Through receiver operating characteristic analysis of the brain volumetric data it was possible to discriminate between the JVR-positive and negative AD subjects with reasonable accuracy (sensitivity = 71.4%; specificity = 88.9%; *p* = 0.007).

**Conclusions:**

JVR is associated with intracranial structural changes in MCI and AD patients, which result in increased WB and GM volumes. The neuropathology of this unexpected and counterintuitive finding requires further investigation, but may suggest that JVR retrogradely transmits venous hypertension into the brain and leads to brain tissues swelling due to vasogenic edema.

## Background

Alzheimer's disease (AD), the most common form of dementia in the elderly, is thought to be caused by an imbalance between amyloid-β (Aβ) production and clearance leading to Aβ accumulation in the central nervous system (CNS), which causes neuronal damage and death, manifesting as progressive clinical dementia [1-3]. It has been shown that patients with AD have 30% slower clearance of Aβ [1]. One of the possible etiologies of decreased Aβ clearance is impaired cerebrospinal fluid (CSF) flow [1,4]. When venous hypertension occurs in the superior sagittal sinus, CSF absorption is also impaired, leading to altered CSF outflow [5-7]. Jugular venous reflux (JVR) characterized by a retrograde flow in internal jugular veins (IJVs) during Valsalva-like manoeuvres (VM) or spontaneously, is found more frequently in the elderly [8,9]. Studies have shown that JVR can retrogradely transmit hypertension into the cerebral venous system and that it is associated with white matter (WM) abnormalities in the elderly [10,11]. Consequently, there is reason to believe that JVR might alter CSF absorption, and that this in turn might influence the clearance of Aβ. Given this, we hypothesized that JVR might be associated with mild cognitive impairment (MCI) and AD, and that this association might manifest itself in structural changes in the brain parenchyma. To this end, we undertook a case-controlled study to explore the issue by evaluating the relationship between JVR and global and tissue specific brain parenchyma volumetric parameters. Volumetric analysis of brain parenchyma structures measured on serial magnetic resonance imaging (MRI) scans has been shown to provide an objective and quantitative method for examining neuropathological changes associated with AD [12-18].

## Methods

### Patient population

Between December 2008 and April 2010, Taiwanese residents consecutively admitted to a memory clinic at Taipei Veterans General Hospital, Taiwan due to subjective memory complaints were assessed for inclusion in this study. Neurologists performed clinical and neurologic evaluations of all participants. Standard neuropsychological assessments, including the Mini-Mental State Examination (MMSE) and Clinical Dementia Rating (CDR) scale were used.

Subjects eligible for participation in the current study were 55 years of age or older, had a CDR score ≤1 (as an assurance that they could cooperate during the Valsalva manoeuvre for JVR detection), and were willing to receive brain MRI and neck duplex ultrasonography. Exclusion criteria for all subjects were a past history of stroke, ischemic heart disease, congestive heart disease, valvular heart disease, cardiac arrhythmia, pulmonary diseases, or malignancy, and having brain MRI of insufficient quality for performing quantitative brain volumetric analysis. The inclusion/exclusion criteria, clinical evaluation, and duplex ultrasonography and MRI protocols and rating method, were pre-defined before the study.

Vascular risk factors were defined according to international guidelines and prospectively identified using all available information including medical charts, laboratory results, patient interviews, and neurological examinations. Hypertension was defined as a history of hypertension, use of antihypertensive medications, or a measured blood pressure consistently >140/90 mmHg. Hyperlipidemia was defined as a cholesterol level >200 mg/dL, low density lipoprotein >150 mg/dL, triglyceride level >150 mg/dL, or history of hyperlipidemia. Diabetes was defined as a history of diabetes, use of medications for diabetes, or an elevated fasting blood glucose >126 mg/dL.

Subjects were classified in AD, mild cognitive impairment (MCI) or control groups according to the criteria of National Institute of Neurological and Communicative Disorders and Stroke/Alzheimer's Disease and Related Disorders Association [19], and by Petersen et al.’s study revised by the Stockholm consensus group [20,21]. The hospital’s Institutional Review Board approved the study and each included participant or his/her caregiver provided informed consent.

### Color-coded duplex ultrasonography for JVR determination

Neck color-coded duplex sonography was performed in all subjects with a 7-MHz linear transducer (iU22; Philips, New York, NY, USA) by the same technician, who was blinded to subjects’ characteristics. On examination, subjects were in a head-straight, flat supine position after a quiet 10 min rest. The IJV was initially insonated longitudinally and thoroughly from the proximal part of the neck base rostrally to the distal part at the submandibular level in order to detect any possible spontaneous JVR at baseline. Then, the VM was performed by forcible expiration from subject’s mouth into a flexible rubber tube connected to a manometer. Subjects were asked to reach 40 mmHg Valsalva pressure and maintain it for at least 10 seconds. During the VM, the distal margin of the window of the color signal was placed at the tip of the flow divider of the internal carotid artery. The color box was adjusted to include the entire lumen of the IJV; if retrograde color appeared in the center of the lumen, the retrograde flow would then be confirmed by Doppler spectrum. JVR was determined when the retrograde-flow color in the center of the lumen and the Doppler-flow waveform demonstrated reversal of flow for more than 0.5 seconds [9-11]. JVR was deemed to have occurred if it could be detected spontaneously at baseline or during the VM. The subjects were classified according to JVR status: subjects with no JVR on both sides were classified as JVR-negative, and subjects with JVR detected on either or both sides during VM, or spontaneously, were graded as being JVR-positive.

### MRI acquisition

All study participants were scanned on 1.5 T MRI (Excite II; GE Medical Systems, Milwaukee, WI). MRI brain sequences included axial two dimensional (2D) T2-weighted imaging (WI) and fluid-attenuated inversion recovery (FLAIR), and three dimensional (3D) T1 spoil gradient echo (SPGR) pulse sequences. The images were acquired with the following sequence parameters: for 3D T1 the repetition time (TR) was 8.545 ms, echo time (TE) 1.844 ms, inversion time (TI) 400 ms, flip angle (FA) 15, slice thickness 1.5 mm, field of view (FOV) 260×260 mm and matrix 256×256; for T2 the TR was 3,700 ms, TE 102 ms, echo train length 19, slice thickness 3 mm, FOV 240×240 mm and matrix 256×256; for FLAIR: TR 9,000 ms, TE 120 ms, TI 2,250 ms, slice thickness 3 mm, FOV 240×240 mm and matrix 256×256.

### MRI analysis

The MRI volumetric analyses were blinded to the subjects’ demographic and clinical characteristics. For brain extraction and tissue segmentation into normalized whole brain (WB), gray matter (GM), WM, and CSF volumes, the SIENAX cross-sectional software tool was used (version 2.6), with corrections for T1-hypointensity misclassification using an in-house developed in-painting program, as previously described [22].

### Statistical analysis

Statistical analysis was undertaken using a combination of the Statistical Package for Social Sciences (SPSS, IBM, Armonk, New York, USA) and in-house algorithms written in Matlab (Mathworks, Natick, Mass) with the aim of evaluating the impact of JVR on the respective MRI variables.

Parametric (one-way ANOVA) and non-parametric (2-tailed Mann Whitney U-test, chi square test) univariate analyses were performed on the respective study cohorts to identify significant differences between the various groups. Values of p < 0.05 were considered statistically significant. For the purposes of this analysis, individuals were simply classified according to clinical disease classification (i.e. controls, MCI and AD) and whether or not they were JVR-positive.

In order to calculate sensitivity and specificity scores related with any structural MRI changes that might be associated with JVR, we also performed receiver operating characteristic (ROC) analysis using a bespoke Matlab algorithm [23]. So as to maximize the discrimination characteristics of the ROC analysis, principal component analysis (PCA) was used to combine MRI variables identified as being influential by the univariate analysis. The ROC analysis was then performed using the first principal component (i.e. the principal component responsible for most variance in the data).

## Results

### Subjects

Eighty-four subjects [57–93 years of age; mean (SD): 79.77 (7.41) years; 33 women; 31 control subjects, 33 MCI subjects, and 20 AD subjects] with eligible brain MRI scans were enrolled according to our criteria. To match age and gender in each disease group, 67 subjects [64–93 years of age; mean (SD): 81.5 (5.3) years; 25 women] were enrolled for further analysis. This study population comprised; 18 control subjects [mean (SD): 81.5 (3.4) years; 4 women], 33 MCI subjects [mean (SD): 81.4 (6.1) years; 13 women], and 16 AD subjects [mean (SD): 81.9 (5.8) years; 8 women] (Table 1). All the AD patients met the diagnostic criteria for AD and CDR = 1; and all MCI patients met the diagnostic criteria for MCI and had a CDR = 0.5. Twenty-nine subjects (43.3%) had right-sided JVR (all detected during VM) and 32 subjects (47.7%) had left-sided JVR (10 detected spontaneously at rest and 22 detected during VM). There were 22 subjects (32.8%) with bilateral JVR; among them, 5 had unilateral spontaneous JVR at rest with contralateral VM-induced JVR and 17 had bilateral VM-induced JVR. Twenty-three subjects (34.3%) presented with JVR on neither side. In the control group, 13 subjects (72.2%) were JVR-positive, and in the MCI group, 24 subjects (72.7%) JVR-positive. By comparison, in the AD group only 43.8% of the subjects (7 subjects) were JVR-positive.

**Table 1:** Demographic and clinical characteristics of study cohort grouped by clinical disease status

**Variable**	**Controls**	**MCI**	**AD**	**Significance p value**
Number of subjects, n (%)	18 (26.9)	33 (49.3)	16 (23.9)	n.a.
Female gender, n (%)	4 (22.2)	13 (39.4)	8 (50.0)	0.233*
Age in years, mean (SD)	81.5 (3.4)	81.4 (6.1)	81.9 (5.8)	0.946
Years of education, mean (SD)	13.5 (1.9)	11.3 (4.1)	10.0 (3.8)	0.018
JVR positive, n (%)	13 (72.2)	24 (72.7)	7 (43.8)	0.106*
MMSE, mean (SD)	28.0 (1.5)	26.2 (1.9)	20.3 (2.8)	<0.001
Hypertension, n (%)	12 (66.7)	17 (51.5)	11 (68.8)	0.401*
Diabetes, n (%)	3 (16.7)	6 (18.2)	3 (18.8)	0.986*
Hyperlipidemia, n (%)	3 (16.7)	3 (0.9)	7 (43.8)	0.015*
Smoking, n (%)	1 (0.6)	1 (3.0)	0 (0.0)	0.637*
Normalized WB volume, mean (SD)	1342.1 (50.6)	1308.9 (62.1)	1291.6 (51.3)	0.034
Normalized GM volume, mean (SD)	728.1 (35.7)	715.3 (44.1)	698.0 (37.5)	0.104
Normalized WM volume, mean (SD)	614.1 (27.7)	593.6 (34.3)	593.6 (29.7)	0.072
CSF volume	383.4 (47.0)	358.1 (60.1)	372.7 (72.9)	0.346

*MCI* Mild cognitive impairment, *AD* Alzheimer’s disease, *JVR* Jugular venous reflux, *MMSE* Mini-mental state examination, *WB* Whole brain, *GM* Gray matter, *WM* White matter, *CSF* Cerebrospinal fluid.

All volumes are expressed in milliliters.

*ANOVA* One-way analysis of variance, *n.a.* Not applicable, *SD* Standard deviation, *n* Number, % - Percentage.

p value determined by one-way ANOVA unless otherwise stated.

*p value determined using chi square test.

### Demographic and clinical univariate analysis

Table 1 shows the comparisons of clinical characteristics and MRI variables between control, MCI and AD groups. From this it can be seen that for all but three of the clinical variables there was no significant difference between the respective groups. The only exceptions to this were: the MMSE score, which was significantly lower in the AD group (*p* < 0.001); the number of years in education, which was on average approximately 3 years less in the MCI and AD groups (*p* = 0.018); and hyperlipidemia, which had a higher incidence in the AD group (*p* = 0.015). Of the MRI variables, only normalized WB volume showed a significant difference between the three groups, being significantly smaller in the AD group (*p* = 0.034).

Table 2 shows demographic, clinical and MRI characteristics of the whole study population aggregated together and grouped according to JVR status (i.e. JVR-positive and negative). The two JVR-graded groups were closely age-matched and had similar clinical characteristics, with no significant differences in sex, education, and disease classification. However, significantly increased normalized WB (*p* = 0.014) and GM (*p* = 0.002) volumes were observed in the JVR-positive group. The increase in brain parenchyma volume in the JVR-positive subjects was matched by a corresponding decrease in CSF volume, although this did not reach significance.

**Table 2:** Demographic and clinical characteristics of study cohort grouped by JVR status (i.e. positive or negative) for all groups aggregated together

**Variable**	**JVR negative**	**JVR positive**	**Significance p value**
Number of subjects, n (%)	23 (34.3)	44 (65.7)	n.a.
Female gender, n (%)	9 (39.1)	16 (36.4)	0.824*
Age in years, mean (SD)	81.6 (3.3)	81.5 (6.2)	0.740
Years of education, mean (SD)	11.8 (3.8)	11.5 (3.7)	0.720
Disease classification, n (%)			0.106*
Control	5 (21.7)	13 (29.5)	
MCI	9 (39.1)	24 (54.5)	
AD	9 (39.1)	7 (15.9)	
MMSE, mean (SD)	24.0 (4.1)	26.0 (3.1)	0.065
Hypertension, n (%)	12 (52.2)	28 (63.6)	0.364*
Diabetes, n (%)	6 (26.1)	6 (13.6)	0.207*
Hyperlipidemia, n (%)	5 (21.7)	8 (18.2)	0.727*
Smoking, n (%)	0 (0.0)	2 (4.5)	0.299*
Normalized WB volume, mean (SD)	1286.6 (58.3)	1327.9 (54.8)	0.014
Normalized GM volume, mean (SD)	692.6 (41.0)	726.1 (40.0)	0.002
Normalized WM volume, mean (SD)	594.0 (32.6)	601.8 (32.5)	0.531
CSF volume	380.5 (58.7)	362.0 (60.9)	0.253

*MCI* Mild cognitive impairment, *AD* Alzheimer’s disease, *JVR* Jugular venous reflux, *MMSE* Mini-mental state examination, *WB* Whole brain, *GM* Gray matter, *WM* White matter, *CSF* Cerebrospinal fluid.

All volumes are expressed in milliliters.

*n.a.* Not applicable, *SD* Standard deviation, *n* Number, % - Percentage.

p value determined by 2-tailed Mann Whitney U-test unless otherwise stated.

*p value determined using chi square test.

In order to determine whether or not the increase in WB and GM volumes was exhibited in all three clinical groups, we repeated the univariate analysis for each disease classification group. The results of this analysis are presented in Table 3, which reveals a trend towards increased WB and GM volumes in the JVR-positive subjects in both the MCI and AD groups, evidenced by Cohen’s d effect sizes >0.8. When the MCI and AD subjects were aggregated together the univariate analysis revealed even more significant increases in both normalized WB (*p* = 0.009) and GM (*p* = 0.003) volumes for the JVR-positive group. No corresponding difference was observed between the JVR-positive and negative subjects in the control group. Similarly in the controls, no significant difference in CSF volume was observed between the JVR-positive and negative groups, whereas in the MCI and AD subjects there was a trend towards reduced CSF volume.

**Table 3:** MRI variables classified according to JVR status (i.e. positive or negative) for each disease group

	**JVR negative**	**JVR positive**	**Significance p value**	**Cohen’s d**
Controls, n (%)	5 (21.7)	13 (29.5)	n.a.	n.a.
Normalized WB volume, mean (SD)	1341.7 (44.6)	1342.3 (54.5)	0.924	0.013
Normalized GM volume, mean (SD)	719.7 (42.2)	731.3 (34.2)	0.633	0.326
Normalized WM volume, mean (SD)	622.0 (23.7)	611.0 (29.4)	0.443	0.396
CSF volume	364.1 (33.5)	390.7 (50.4)	0.336	0.566
MCI, n (%)	9 (39.1)	24 (54.5)	n.a.	n.a.
Normalized WB volume, mean (SD)	1274.9 (68.3)	1321.7 (55.8)	0.079	0.754
Normalized GM volume, mean (SD)	686.8 (49.3)	726.0 (37.7)	0.045	0.890
Normalized WM volume, mean (SD)	588.1 (32.8)	595.7 (35.3)	0.824	0.221
CSF volume	377.3 (54.2)	350.8 (61.7)	0.284	0.440
AD, n (%)	9 (39.1)	7 (15.9)	n.a.	n.a.
Normalized WB volume, mean (SD)	1267.8 (35.2)	1322.2 (54.7)	0.054	1.060
Normalized GM volume, mean (SD)	683.4 (26.6)	716.8 (42.9)	0.142	0.889
Normalized WM volume, mean (SD)	584.4 (30.4)	605.5 (26.2)	0.252	0.709
CSF volume	392.9 (75.1)	346.8 (66.2)	0.252	0.632
MCI and AD combined, n (%)	18 (78.3)	31 (70.5)	n.a.	n.a.
Normalized WB volume, mean (SD)	1271.4 (52.8)	1321.8 (54.7)	0.009	0.858
Normalized GM volume, mean (SD)	685.1 (38.4)	723.9 (38.4)	0.003	0.914
Normalized WM volume, mean (SD)	586.3 (30.8)	597.9 (3.34)	0.356	0.358
CSF volume	385.1 (64.0)	349.9 (61.6)	0.076	0.548

*MCI* Mild cognitive impairment, *AD* Alzheimer’s disease, *JVR* Jugular venous reflux, *WB* Whole brain, *GM* Gray matter, *WM* White matter, *CSF* Cerebrospinal fluid.

Volumes are expressed in millilitres.

*n.a.* not applicable, *n* Number, *SD* Standard deviation, % - Percentage.

p values determined by 2-tailed Mann Whitney U-test.

Separate analysis of the JVR-negative group revealed a statistically significant difference between the controls and the MCI and AD subjects for the normalized WB (*p* = 0.023) and WM (*p* = 0.028) volumes, both of which were greatly reduced in the JVR-negative MCI and AD subjects. By comparison, no corresponding reductions in brain parenchyma volume were observed in the JVR-positive MCI and AD subjects compared with the JVR-positive controls.

### Receiver operating curve analysis

**Figure 1:**
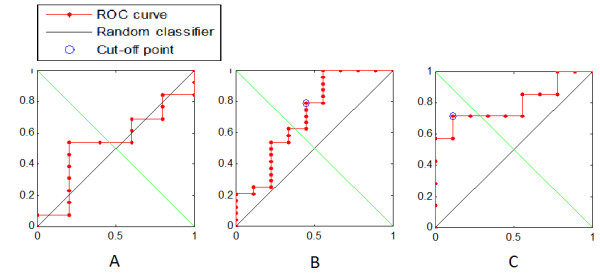
**Receiver operating characteristic (ROC) curves for: (A) the controls; (B) the MCI subjects; (C) the AD patients.** ROC undertaken using the first principal component of the normalized whole brain and gray matter volumes combined.

The results of the univariate analysis revealed JVR to be associated with a trend towards increased WB and GM volumes in both the MCI and AD groups, something that was not observed in the control group. In order to confirm this finding we used PCA to orthogonalize/combine these variables and used the resulting first principal component to perform a ROC analysis, the results of which are presented in Figure 1 and Table 4. From this, it can be seen that the ROC results are strongly significant for the MCI and AD groups, and appear to corroborate the findings of the univariate analysis. While the ROC analysis did not yield a significant result for the control group, it was able to discriminate between the JVR-positive and negative subjects in the other two groups with reasonable accuracy (>70%). Indeed, for the AD group, the ROC analysis achieved sensitivity and specificity scores of 71.4% and 88.9%, respectively (*p* = 0.007). As such, the results suggest that JVR was associated with structural changes in the brain parenchyma in both the MCI and AD subjects.

**Table 4:** Results of receiver operating curve (ROC) analysis of jugular venous reflux status using the first principal component of the normalized whole brain and gray matter volumes combined

**Group**	**Area under curve**	**True positives**	**False negatives**	**True negatives**	**False positives**	**Sensitivity**	**Specificity**	**Accuracy**	**Significance p value**
Controls	0.538	n.s.	n.s.	n.s.	n.s.	n.s.	n.s.	n.s.	0.401
MCI	0.713	19	5	5	4	79.2%	55.5%	72.7%	0.012
AD	0.794	5	2	8	1	71.4%	88.9%	81.3%	0.007

*MCI* Mild cognitive impairment, *AD* Alzheimer’s disease.

*n.s.* Not significant.

## Discussion

The results of the study do not support the hypothesis that JVR is specifically associated with MCI and AD. The incidence of JVR was very similar in both the control and MCI groups, and was actually lower in the AD group. Having said this, the results suggest that JVR is associated with a rather unexpected phenomenon. JVR appears to be associated with structural changes in the brain parenchyma of patients with MCI and AD that were not observed in the control group. This is highlighted in the results presented in Table 3, which revealed a marked difference in response to JVR between the controls and the other two groups. Overall, the subjects with JVR had greater WB and GM volumes, which was accompanied by decreased CSF volume, compared with those without JVR (Table 2). This effect was particularly marked in the MCI and AD groups, whereas it was absent in the controls (Table 3). As such, the observation that AD patients with JVR exhibit larger WB volumes is a surprising finding, as AD is normally characterized by advanced brain atrophy. This can be clearly seen if one analyzes the JVR-negative and positive groups separately. In the JVR-negative subjects there was a statistically significant reduction in global and tissue specific brain parenchymal volumes in the AD and MCI groups compared with the controls - a finding that is consistent with the observations of many other researchers [12-15,24]. However, no corresponding reduction was observed in the JVR-positive group, implying that in some way JVR inhibited brain volumetric loss in the AD and MCI subjects. Compared with insufficient cerebral arterial supply (arterial ischemia), cerebral venous drainage impairment with venous hypertension causes more severe vasogenic edema and brain–blood barrier damage [5-7]. Previous studies of JVR provide evidence that retrograde-transmitted venous hypertension from JVR can reach the cerebral venous system [10,11]. It is therefore possible that JVR retrogradely transmits venous hypertension into the brain, leading to increased permeability of the blood–brain barrier (BBB), resulting in vasogenic edema, causing the brain tissue to swell. Disruption of the BBB will allow plasma molecules to pass into the brain, with the result that an osmotic pressure gradient is established which will contribute to edema formation. While it is not known if this mechanism is at work, it is noticeable that JVR was associated with a marked reduction in CSF volume in the MCI and AD subjects, something that would be consistent with an influx of CSF into the parenchymal tissue. Alternatively, JVR might promote the retention of blood in the cerebral veins [25] – something that might increase the volume of the brain parenchyma.

The results of the ROC analysis demonstrate that it is possible to discriminate between the JVR-positive and negative MCI and AD subjects with reasonable accuracy using just the MRI variables, normalized WB volume and normalized GM volume, whereas this was not the case in the control group. As such, this finding appears to corroborate those of the univariate analysis. Furthermore, the ROC analysis suggests that a progressive effect may be occurring, which is stronger in the AD group (area under curve (AUC) = 0.794; *p* = 0.007) than in the MCI group (AUC = 0.713; *p* = 0.012). While this finding is difficult to interpret, it is known that MCI is frequently a precursor to AD [26].

While the exact physiological mechanisms behind our intriguing observations are unclear, it is known that JVR can induce hypertension in the dural sinuses [10,11] and that this can alter intracranial CSF dynamics [27]. Therefore, it may be that retrograde-transmitted venous pressure associated with JVR inhibits CSF absorption into the superior sagittal sinus [28]. Absorption of CSF into the dural venous sinuses requires a pressure gradient of about 5–7 mmHg [29,30]. Therefore, an increase in venous pressure of few mmHg due to occlusion of the venous drainage pathways [31], or reflux, will tend to inhibit the bulk flow of CSF, as observed by Zamboni et al. [32]. If CSF flow is inhibited, then this might result in increased biochemical concentrations in the CSF. Overproduction of Aβ is thought to damage WM in AD [33]. Given that patients with AD have been shown to exhibit 30% slower Aβ clearance [1], it has been postulated [4] that accumulation of Aβ in the CSF, arising from venous hypertension, may precipitate the onset of AD. However, our results do not support this conclusion. While JVR may be associated with accumulation of Aβ in the CSF, our results do not indicate that JVR precipitates the onset of AD. Indeed, the majority of AD patients in our study were JVR-negative. Having said this, the results suggest that JVR is having an effect on the brain parenchyma of the MCI and AD patients, and the possibility that this might involve CSF accumulation of Aβ cannot be excluded. Further investigations are therefore needed to elucidate the underlying neuropathological mechanisms associated with our observations.

In this study we focused solely on JVR and ignored other phenomena associated with constricted cerebral venous outflow [34]. However, it may be that restricted venous outflow, such as that associated with chronic cerebrospinal venous insufficiency [35,36], might also be influential. Studies of cerebral arteriovenous malformation have shown that the elevated venous pressure and its insults to intracranial structures are more severe when combined with obstruction in other venous outflow tracts [37,38]. Therefore it may be the case in JVR, that retrogradely-transmitted venous pressure into cerebral circulation needs additionally an obstruction of contralateral venous outflow pathway to cause significant venous hypertension and consequently intracranial insults. It is important to remember that a diagnosis of ‘no-JVR’ does not preclude the possibility that constricted cerebral venous outflow might be present. Furthermore, engorged veins are frequently observed upstream of stenotic lesions [39] and it may be that muscular compression of these veins also contributes to JVR.

Although it yielded novel and interesting findings, it should be noted that our study was limited in its scope, having a relatively small sample size. In particular, the AD group contained fewer JVR-positive individuals compared with the other two groups. Also, because of the limited numbers involved, we restricted ourselves to a simple positive/negative JVR classification and did not distinguish between bilateral, left and right-sided JVR. It is therefore not known the extent to which left and right sidedness in JVR influences brain atrophy and further work will be required to evaluate this. Furthermore, there are other venous abnormalities associated with aging and other neurological disorders which we were not able to assess [9,27,40]. Nevertheless, our findings are novel and suggest that cerebral venous drainage impairment may influence the neuropsychology of AD. Further studies, particularly longitudinal studies, are therefore needed to build on our initial findings.

## Conclusions

JVR is associated with intracranial structural changes in MCI and AD patients, which result in increased WB and GM volumes. Although the neuropathology associated with this unexpected and counterintuitive finding requires further investigation, it may be that JVR retrogradely transmits venous hypertension into the brain, and that this leads to the brain tissues swelling due to vasogenic edema.

## Abbreviations

AD: Alzheimer disease; Aβ: Amyloid-β; ANOVA: Analysis of variance; BBB: Blood brain barrier; CDR: Clinical dementia rating; CSF: Cerebrospinal fluid; DS: Doppler sonography; FLAIR: Fluid attenuated inversion recovery; FOV: Field of view; GM: Gray matter; IJV: Internal jugular vein; JVR: Jugular venous reflux; LV: Lateral ventricle; MMSE: Mini-mental state examination; MRI: Magnetic resonance imaging; NPH: Normal pressure hydrocephalus; PCA: Principal component analysis; ROC: Receiver operating characteristic; WM: White matter.

## Competing interests

The authors declare that they have no competing interests regarding study in question. Robert Zivadinov received personal compensation from Teva Pharmaceuticals, Biogen Idec, EMD Serono Claret and Genzyme for speaking and consultant fees. Dr. Zivadinov received financial support for research activities from Biogen Idec, Teva Pharmaceuticals, Genzyme and Novartis. Clive Beggs, Niels Bergsland, Simon Shepherd, Michael Dwyer, Chih-Ping Chung, Pei-Ning Wang and Han-Hwa Hu have nothing to disclose.

## Authors’ contributions

CBB, CPC, NB, PNW, SJS, CYC, MGD, HHH, and RZ have made substantial contributions to conception and design, or acquisition of data, or analysis and interpretation of data. CBB, CPC and RZ have been involved in drafting the manuscript, while NB, PNW, SJS, CYC, MGD and HHH revised it critically for important intellectual content. All authors have given final approval of the version to be published.

## Pre-publication history

The pre-publication history for this paper can be accessed here:

http://www.biomedcentral.com/1471-2377/13/157/prepub

## References

[B1] Mawuenyega KG Sigurdson W Ovod V Munsell L Kasten T Morris JC Yarasheski KE Bateman RJ Decreased clearance of CNS beta-amyloid in Alzheimer's disease Science 2010 330 6012 1774 10.1126/science.1197623 21148344 PMC3073454

[B2] Hardy J Selkoe DJ The amyloid hypothesis of Alzheimer's disease: progress and problems on the road to therapeutics Science 2002 297 5580 353 356 10.1126/science.1072994 12130773

[B3] Cummings JL Alzheimer's disease N Engl J Med 2004 351 1 56 67 10.1056/NEJMra040223 15229308

[B4] Reed-Cossairt A Zhu X Lee HG Reed C Perry G Petersen RB Alzheimer's disease and vascular deficiency: lessons from imaging studies and down syndrome Curr Gerontol Geriatr Res 2012 2012 929734 22400025 10.1155/2012/929734 PMC3286884

[B5] Cutler RW Page L Galicich J Watters GV Formation and absorption of cerebrospinal fluid in man Brain 1968 91 4 707 720 10.1093/brain/91.4.707 5304069

[B6] Schaller B Physiology of cerebral venous blood flow: from experimental data in animals to normal function in humans Brain Res Brain Res Rev 2004 46 3 243 260 10.1016/j.brainresrev.2004.04.005 15571768

[B7] Schaller B Graf R Cerebral venous infarction: the pathophysiological concept Cerebrovasc Dis 2004 18 3 179 188 10.1159/000079939 15273432

[B8] Akkawi NM Agosti C Borroni B Rozzini L Magoni M Vignolo LA Padovani A Jugular valve incompetence: a study using air contrast ultrasonography on a general population J Ultrasound Med 2002 21 7 747 751 12099562 10.7863/jum.2002.21.7.747

[B9] Chung CP Lin YJ Chao AC Lin SJ Chen YY Wang YJ Hu HH Jugular venous hemodynamic changes with aging Ultrasound Med Biol 2010 36 11 1776 1782 10.1016/j.ultrasmedbio.2010.07.006 20800950

[B10] Chung CP Hsu HY Chao AC Cheng CY Lin SJ Hu HH Jugular venous reflux affects ocular venous system in transient monocular blindness Cerebrovasc Dis 2010 29 2 122 129 10.1159/000262307 19955735

[B11] Wu IH Sheng WY Hu HH Chung CP Jugular venous reflux could influence cerebral blood flow: a transcranial Doppler study Acta Neurol Taiwan 2011 20 1 15 21 21249580

[B12] Forstl H Zerfass R Geiger-Kabisch C Sattel H Besthorn C Hentschel F Brain atrophy in normal ageing and Alzheimer's disease. Volumetric discrimination and clinical correlations Br J Psychiatry 1995 167 6 739 746 10.1192/bjp.167.6.739 8829740

[B13] Obara K Meyer JS Mortel KF Muramatsu K Cognitive declines correlate with decreased cortical volume and perfusion in dementia of Alzheimer type J Neurol Sci 1994 127 1 96 102 10.1016/0022-510X(94)90141-4 7699398

[B14] Shear PK Sullivan EV Mathalon DH Lim KO Davis LF Yesavage JA Tinklenberg JR Pfefferbaum A Longitudinal volumetric computed tomographic analysis of regional brain changes in normal aging and Alzheimer's disease Arch Neurol 1995 52 4 392 402 10.1001/archneur.1995.00540280078021 7710375

[B15] Sullivan EV Shear PK Mathalon DH Lim KO Yesavage JA Tinklenberg JR Pfefferbaum A Greater abnormalities of brain cerebrospinal fluid volumes in younger than in older patients with Alzheimer's disease Arch Neurol 1993 50 4 359 373 10.1001/archneur.1993.00540040021009 8460957

[B16] Petrella JR Coleman RE Doraiswamy PM Neuroimaging and early diagnosis of Alzheimer disease: a look to the future Radiology 2003 226 2 315 336 10.1148/radiol.2262011600 12563122

[B17] Ott BR Cohen RA Gongvatana A Okonkwo OC Johanson CE Stopa EG Donahue JE Silverberg GD Alzheimer's Disease Neuroimaging I Brain ventricular volume and cerebrospinal fluid biomarkers of Alzheimer's disease J Alzheimers Dis 2010 20 2 647 657 20182051 10.3233/JAD-2010-1406 PMC3078034

[B18] Nestor SM Rupsingh R Borrie M Smith M Accomazzi V Wells JL Fogarty J Bartha R Ventricular enlargement as a possible measure of Alzheimer's disease progression validated using the Alzheimer's disease neuroimaging initiative database Brain 2008 131 Pt 9 2443 2454 18669512 10.1093/brain/awn146 PMC2724905

[B19] McKhann G Drachman D Folstein M Katzman R Price D Stadlan EM Clinical diagnosis of Alzheimer's disease: report of the NINCDS-ADRDA Work Group under the auspices of Department of Health and Human Services Task Force on Alzheimer's Disease Neurology 1984 34 7 939 944 10.1212/WNL.34.7.939 6610841

[B20] Petersen RC Doody R Kurz A Mohs RC Morris JC Rabins PV Ritchie K Rossor M Thal L Winblad B Current concepts in mild cognitive impairment Arch Neurol 2001 58 12 1985 1992 10.1001/archneur.58.12.1985 11735772

[B21] Artero S Petersen R Touchon J Ritchie K Revised criteria for mild cognitive impairment: validation within a longitudinal population study Dement Geriatr Cogn Disord 2006 22 5–6 465 470 17047325 10.1159/000096287

[B22] Zivadinov R Heininen-Brown M Schirda CV Poloni GU Bergsland N Magnano CR Durfee J Kennedy C Carl E Hagemeier J Abnormal subcortical deep-gray matter susceptibility-weighted imaging filtered phase measurements in patients with multiple sclerosis: a case–control study Neuroimage 2012 59 1 331 339 10.1016/j.neuroimage.2011.07.045 21820063

[B23] Cardillo G ROC curve: compute a Receiver Operating Characteristics curve [http://www.mathworks.com/matlabcentral/fileexchange/19950-roc-curve]

[B24] Leung KK Bartlett JW Barnes J Manning EN Ourselin S Fox NC Cerebral atrophy in mild cognitive impairment and Alzheimer disease: Rates and acceleration Neurology 2013 80 7 648 654 10.1212/WNL.0b013e318281ccd3 23303849 PMC3590059

[B25] Kitano M Oldendorf WH Cassen B The Elasticity of the Cranial Blood Pool J Nucl Med 1964 5 613 625 14212187

[B26] Shah Y Tangalos EG Petersen RC Mild cognitive impairment. When is it a precursor to Alzheimer's disease? Geriatrics 2000 55 9 62, 65–68 10997127

[B27] Beggs CB Venous Haemodynamics in Neurological Disorders: An Analytical Review with Hydrodynamic Analysis BMC Medicine. BMC Med 2013 11 142 10.1186/1741-7015-11-142 PMC3668302 23724917

[B28] Ekstedt J CSF hydrodynamic studies in man. 2. Normal hydrodynamic variables related to CSF pressure and flow J Neurol Neurosurg Psychiatry 1978 41 4 345 353 10.1136/jnnp.41.4.345 650242 PMC493028

[B29] McCormick JM Yamada K Rekate HL Miyake H Time course of intraventricular pressure change in a canine model of hydrocephalus: its relationship to sagittal sinus elastance Pediatr Neurosurg 1992 18 3 127 133 10.1159/000120650 1457371

[B30] Olivero WC Rekate HL Chizeck HJ Ko W McCormick JM Relationship between intracranial and sagittal sinus pressure in normal and hydrocephalic dogs Pediatr Neurosci 1988 14 4 196 201 10.1159/000120388 3269540

[B31] Zamboni P Galeotti R Menegatti E Malagoni AM Gianesini S Bartolomei I Mascoli F Salvi F A prospective open-label study of endovascular treatment of chronic cerebrospinal venous insufficiency J Vasc Surg 2009 50 6 1348 1358 e1341-1343 10.1016/j.jvs.2009.07.096 19958985

[B32] Zamboni P Menegatti E Weinstock-Guttman B Schirda C Cox JL Malagoni AM Hojanacki D Kennedy C Carl E Dwyer MG The severity of chronic cerebrospinal venous insufficiency in patients with multiple sclerosis is related to altered cerebrospinal fluid dynamics Funct Neurol 2009 24 3 133 138 20018140

[B33] Querfurth HW LaFerla FM Alzheimer's disease N Engl J Med 2010 362 4 329 344 10.1056/NEJMra0909142 20107219

[B34] Zamboni P Galeotti R Menegatti E Malagoni AM Tacconi G Dall'Ara S Bartolomei I Salvi F Chronic cerebrospinal venous insufficiency in patients with multiple sclerosis J Neurol Neurosurg Psychiatry 2009 80 4 392 399 19060024 10.1136/jnnp.2008.157164 PMC2647682

[B35] Zamboni P Menegatti E Conforti P Shepherd S Tessari M Beggs C Assessment of cerebral venous return by a novel plethysmography method J Vasc Surg 2012 56 677 685 10.1016/j.jvs.2012.01.074 22521804

[B36] Beggs C Shepherd S Zamboni P Cerebral venous outflow resistance and interpretation of cervical plethysmography data with respect to the diagnosis of chronic cerebrospinal venous insufficiency Phlebology 2012 doi:10.1258/phleb.2012.012039:1–9 10.1258/phleb.2012.012039 23060482

[B37] Fierstra J Conklin J Krings T Slessarev M Han JS Fisher JA Terbrugge K Wallace MC Tymianski M Mikulis DJ Reply: A comment on impaired peri-nidal cerebrovascular reserve in seizure patients with brain arteriovenous malformations Brain 2012 135 1 2 10.1093/brain/awr346 21109501

[B38] Deguchi J Yamada M Kobata H Kuroiwa T Regional cerebral blood flow after acetazolamide challenge in patients with dural arteriovenous fistula: simple way to evaluate intracranial venous hypertension AJNR Am J Neuroradiol 2005 26 5 1101 1106 15891167 PMC8158625

[B39] Zamboni P Consorti G Galeotti R Gianesini S Menegatti E Tacconi G Carinci F Venous Collateral Circulation Of The Extracranial Cerebrospinal Outflow Routes Curr Neurovasc Res 2009 6 3 204 212 10.2174/156720209788970054 19534716

[B40] Haacke EM Beggs CB Habib C The role of venous abnormalities in neurological disease Rev Recent Clin Trials 2012 7 2 100 116 10.2174/157488712800100305 22338620

